# Immobilized enzymes as potent antibiofilm agent

**DOI:** 10.1002/btpr.3281

**Published:** 2022-06-24

**Authors:** Dibyajit Lahiri, Moupriya Nag, Ankita Dey, Tanmay Sarkar, Rina Rani Ray, Maksim Rebezov, Mohammad Ali Shariati, Muthu Thiruvengadam, Jesus Simal‐Gandara

**Affiliations:** ^1^ Department of Biotechnology University of Engineering & Management Kolkata West Bengal India; ^2^ Department of Microbiology Belle Vue Clinics Kolkata West Bengal India; ^3^ Department of Food Processing Technology, Malda Polytechnic West Bengal State Council of Technical Education, Govtment of West Bengal Malda 732102 India; ^4^ Department of Biotechnology Maulana Abul Kalam Azad University of Technology Haringhata West Bengal India; ^5^ V. M. Gorbatov Federal Research Center for Food Systems Moscow Russian Federation; ^6^ Prokhorov General Physics Institute of the Russian Academy of Sciences Moscow Russian Federation; ^7^ Liaocheng University Liaocheng Shandong China; ^8^ Department of Scientific Research K. G. Razumovsky Moscow State University of Technologies and Management (The First Cossack University) Moscow Russian Federation; ^9^ Department of Crop Science College of Sanghuh Life Science, Konkuk University Seoul South Korea; ^10^ Universidade de Vigo, Nutrition and Bromatology Group Analytical Chemistry and Food Science Department, Faculty of Science Ourense Spain

**Keywords:** antibiofilm, covalent crosslinking, enzyme, gel entrapment, immobilization

## Abstract

Biofilm has been a point of concern in hospitals and various industries. They not only cause various chronic infections but are also responsible for the degradation of various medical appliances. Since the last decade, various alternate strategies are being adopted to combat the biofilm formed on various biotic and abiotic surfaces. The use of enzymes as a potent anti‐fouling agent is proved to be of utmost importance as the enzymes can inhibit biofilm formation in an eco‐friendly and cost‐effective way. The physical and chemical immobilization of the enzyme not only leads to the improvement of thermostability and reusability of the enzyme, but also gains better efficiency of biofilm removal. Immobilization of amylase, cellobiohydrolase, pectinase, subtilisin A and β‐N‐acetyl‐glucosaminidase (DspB) are proved to be most effective in inhibition of biofilm formation and removal of matured biofilm than their free forms. Hence, these immobilized enzymes provide greater eradication of biofilm formed on various surfaces and are coming up to be the potent antibiofilm agent.

## 1 INTRODUCTION

Bacterial biofilms have a manifold harmful effect on human society.^1^ Being the foremost cause of biofouling in most industrial systems, and various life‐threatening issues in the health care sectors, biofilm is responsible for the loss of more than billions of dollars and serious health crises throughout the world. A biofilm is an agglomeration of bacteria on a surface, where the sessile microcolonies dwelling remain encompassed by self‐secreted extracellular polymeric substances (EPS) comprising of carbohydrate, proteins, lipids, nucleic acid, and other minerals that not only provide nourishment to the in‐dwelling cells but also protects the cells from environmental stresses.^2^ The bacterial species possess the property of adhering to the surface in the form of biofilm and forms an important survival strategy within nature.^3^

It can bring about the development of antimicrobial resistance, damage to the equipment, failure in transplant surgery, energy loss, contamination of products, and the onset of various types of chronic infections.^4^ Medical prosthetics like implantable medical devices have become an important part of the modern health care sector providing an enhanced quality of life to millions of people.^5^ But, unfortunately, the success of such implantation is severely hindered due to the formation of biofilm on the abiotic surface of the implant^6^ and such biofilms are difficult to get eradicated.

Moreover, increased amount of morbidity, mortality, enhanced costs in the healthcare sectors, and prolonged period of hospitalization are mostly associated with the medical device associated biofilm infections.^6^

Hence, the dispersal of multispecies biofilms become the need of the hour. The conventional antimicrobial methods have been shown to eradicate planktonic microbes easily but have proved to be ineffective in the removal of sessile microcolonies.^7^ Thus, alternate novel strategies are considered to be essential in fulfilling the requirement of removing the biofilm.

Although the concept of using enzymes to inhibit the formation of unwanted biofilms is not new, the scientific literature still lacks important information about the effects of immobilized biocatalysts and their impacts on biofilm formation as the scientific community underestimated or neglected the impacts of immobilized enzymes on biofilm structure and resistance to traditional antimicrobial agents.^8^

The present review focuses on the elucidation of antibiofilm efficacies of various enzymes, both free and immobilized, their degradation architecture, advantages and instances of immobilized enzyme as antibiofilm agent, use of nanocomposite of immobilized enzymes for treatment of biofilms, mechanism of quorum‐quenching.

### 1.1 Enzymes as antibiofilm and anti‐biofouling agents

Enzymes possess the potential to control the process of biofouling.^9^ by removing various types of biomolecular films and proteins from various biotic and abiotic surfaces.^10^ The enzymes are considered to be an attractive anti‐biofouling agent as they are natural molecules and eco‐friendly in nature due to their easy bio‐degradability. Due to their nontoxic nature and affordable prices, some of these enzymes are effectively used as antifouling paints in the marine environment as a substitute for biocides.^11^

High substrate affinity of the enzymes along with the economic and environmental friendliness resulted in the use of enzymes within detergent formulations for the purpose of the removal of biofilm.^12^ Enzymes are also found to have therapeutic functions in the removal of pathogenic biofilms.^13^ In recent times, a variety of enzymes enriched products have been commercialized that include tablets, rinsing solutions, chewing gums for dental treatment, and denitrifies containing enzymes like lysins, dextranase, mutanase, and so forth that can serve to play an effective role in the disintegration of the biofilm matrix^13^ formed on different parts of the body.

### 1.2 Lysozymes

Lysozymes are the group of hydrolytic enzymes that have shown efficacy in the hydrolysis of the cell wall and have been used to create a coating in most antibacterial agents. The most widely used lysozyme is isolated from hen egg white.^14^ They can cleave the β‐glycosidic bond between N‐acetylmuramic acid of C1 and N‐acetylglucosamine of C4 and destabilize the bacterial cell wall structure. Although Gram‐negative bacteria show resistance to lysozymes due to the presence of an outer membrane that hinders the accessibility of the enzyme to the peptidoglycan,^14^ a large group of Gram‐positive bacterial species is susceptible to lysozyme attack, causing cell lysis. However, the susceptibility of the Gram‐negative bacteria can be enhanced by pre‐treating the cells with detergents, chelating agents like EDTA, or in the presence of high hydrostatic pressure.^14^ The non‐enzymatic mode of lysozyme is based on the amphibolic and cationic properties of the enzyme^15^ results in the perturbation of the plasma membrane and thereby activates the autolytic system within the bacteria.^16^

### 1.3 Autolysins

Autolysins are a group of membrane‐bound enzymes that bring about degradation of the peptidoglycans of the cell wall, resulting in the death of the cells.^17^ Lysostaphin and the catalytic domain of LytM, the pentaglycine endopeptidases are responsible for the cleaving of the crosslinking of the peptidoglycan bridges present in the cell wall of *Staphylococcus* sp.^18^ It is widely used to eradicate susceptible *Staphylococcus aureus* and *Staphylococcus epidermidis* biofilms.^19^

### 1.4 Amylase

Amylase has been also documented to have a potential antibiofilm property by the mechanism of degrading the polysaccharide associated with the biofilm architecture.^7^ The α‐amylase produced extracellularly by *Bacillus subtilis* S8–18 from the marine environment showed its antibiofilm efficacy against methicillin‐resistant *S. aureus*.^20^ It has also been found that enzymes like cellobiose dehydrogenase and amylase act synergistically and showed higher efficacy as antibiofilm and antibacterial agents. The cellobiose dehydrogenase acts upon various types of oligosaccharides and cellulose with the liberation of hydrogen peroxide. They act effectively in the mechanism of eradicating the biofilm. Pectinase also showed a potent enzyme in the mechanism of degrading the adhesive proteins thereby preventing the development of biofilm by *Enterococcus faecalis*, *S. aureus*, and *Pseudomonas aeruginosa*.^21^

### 1.5 Glycoside hydrolase

They are usually produced by a group of opportunistic fungi like Aspergillus fumigatus and Gram‐negative bacterial specie like *P. aeruginosa* that can be used for the purpose of degrading the biofilm formed by fungal species and thereby help in reducing virulence.^22^

Often, commercial biofilm exclusion requires the synergistic action of complex enzymes like proteases, lipases, and amylases that enhances the efficacy of removing complex biofilm from abiotic surfaces.^23^ Various alternative strategies that help in determining the antifouling potential of enzymes comprise screening of the enzymes that help in the cleaving of the specific substrate, helping in cellular adhesion to the surface.^24^ Thus, the process of enzyme screening plays a vital role in the process of targeting single or multiple species of foulers.^25^

The enzymes associated with anti‐biofouling may be equipped with the power of degradation of compounds that can counteract adhesives, degradation of the extracellular polymeric substance (EPS), and denaturation of intercellular communication molecules leading to inhibition of quorum sensing (QS).^26^

## 2 ENZYMES AFFECTING DIFFERENT EVENTS OF BIOFILM FORMATION

### 2.1 Cell lysis by enzymes

Various types of lytic enzymes are found to act as antibacterial agents and can release cellular components like proteins and DNA^27^ by degradation of the bacterial cell wall with the help of various hydrolytic enzymes. These cell‐lysing hydrolytic enzymes can be classified as glycosidases (for cleavage of the polysaccharide chains), endopeptidases (for cleavage of the polypeptide chains), and amidases (for cleavage of peptides and polysaccharides).^28^, ^29^ Noteworthy lytic enzymes are: murein hydrolase, a glycosidase enzyme produced by plants and animals part of their defense system, endolysins, produced by bacteriophages and microlysins, produced by most microbes apart from bacteriophages.^28^

### 2.2 Degradation of biofilm architecture

Extracellular polymeric substance (EPS) being a complex matrix comprising mainly of carbohydrates, proteins, fats, nucleic acids is the primary target of enzymes^30^ and enzymes by degrading the EPS can bring about the dispersion of the biofilm. The enzymes exert their antibiofilm efficacies by degrading the EPS, followed by reducing the mechanical stability of the biofilm.^2^ This results in the easy exposure of the sessile communities to antimicrobial agents and antibiotics causing an enhancement in its activity. Enzymes like dispersin B and protease action on the biofilm formed by *S. aureus* result in the elimination of the biofilm. Dispersin B possesses the ability to bring about the degradation of N‐acetyl‐D‐glucosamine residues^31^ and degrade the biofilm formed by *S. epidermidis* and *S. aureus*.^32^ Cellulase has been found to have efficacy in biofilm removal in the textile, paper, and food industries.^33^ Biofilm formed by *P. aeruginosa* is found to be affected in the presence of cellulase. Enzymes have shown their efficacy in removal of the dental plaque infections by bringing about the degradation of the structural components of the plaque.^34^ Glycoside hydrolase β‐N‐acetylhexosaminidase (or dispersin B) is another very useful enzyme that has shown its efficacy in inhibiting the biofilm formed by major group of bacterial cells.^35^

### 2.3 Enzymatic degradation of adhesives being produced by the sessile colonies

The adhesion of the bacterial colonies on the biotic and abiotic surfaces is mediated by various types of proteins or glycoproteins and various types of polysaccharides that help in the effective adhesion to the surface.^36^ Thus, different enzymes play an effective role in the process of degrading various chemical components, facilitating bacterial adhesions, and thereby preventing the process of biofouling.^37^ Various commercially used enzymes like hydrolase, lipases, and proteases prevent the setting of the microbial cells on the surface and degrade the adhesive components. Two important mechanisms that are responsible for the disintegration of the adhesive polymers as well as proteins that help in the surface attachment.^38^

### 2.4 Chemical characteristics of enzymes making it a potent antibiofilm/antimicrobial agent

The chemical attributes of the enzymes responsible for antibiofilm activity have a potent site of action like that of the matrix of the biofilm. Enzymes like lysostaphin, beta‐*N*‐acetylglucosaminidase, DNase I, and dispersin B possess the ability to prevent the adhesion by the sessile microbial communities.^39^ Studies have revealed that combinatorial activity of the enzymes dispersin B and DNase I possess the ability to inhibit colonization by *S. aureus*.^40^ The enzymes possess the ability to bring about degradation of various types of polysaccharides, eDNA, proteins, and various other types of QS molecules.^41^ The various types of enzymes also possess the ability to bring about hydrolysis of various types of autoinducers like acylases, lactonases, and oxidoreductase enzymes.^42^ The ability of body to combat against microbial species includes the production of large amounts of superoxides those are associated with the membrane associated NADPH oxidase. Xanthine oxidase, cyclooxygenases, and lipoxygenases are responsible for the production of superoxide anion.^43^ The superoxides are the group of reactive oxygen species which acts as a source for the production of other ROS.^44^ The enzyme superoxide dismutases into hydrogen peroxides that are being used for destroying the invading pathogens. Peroxidases like myeloperoxidases and lactoperoxidases use the hydrogen peroxides for the purpose of oxidizing the halides thereby bringing about reduction in the pathogenic organisms and act as potent antibiofilm and antimicrobial agent.

### 2.5 Enzyme‐mediated mechanism of quorum‐quenching

The sessile microcolonies communicate with each other by a density‐dependent cellular communication mechanism known as QS.^45^ Various enzymes can obstruct such communication (Table 1) by degrading the QS signal molecules and thereby can prevent the formation of biofilm.^46^ Since, Gram‐negative bacteria communicate by acylhomoserine lactone (AHL) whereas Gram‐positive bacteria by autoinducer peptides,^45^ the enzymes required for hindering QS in them are different. AHL acylase breaks the amide bonds present within the acyl chains of the homoserine lactone rings^47^ while adenine dinucleotide phosphate oxidase can inactivate the autoinducer peptide signals through oxidation of the C‐terminal methionine, associated with the peptides.^48^

**TABLE 1:** Enzymes with anti‐QS activities

Name of the enzyme	Source	Function	Reference
AHL Lactonase	Produced from wide varieties of plants, fungi, bacteria and algae	Helps in the breakdown of the HSL ring	73
AHL oxidoreductase	Produced from wide varieties of plants, fungi, bacteria and legumes	It brings about degradation of the acyl chain of HSL by oxidation or reduction	73
AHL‐acylase	Produced from wide varieties of plants, fungi, bacteria and legume	It brings about hydrolysis of amide linkage and degradation of HSL	73
2‐Alkyl‐3‐hydroxy4(1H)‐quinolone 2,4‐dioxygenase	*Arthrobacter* sp	Inhibits the QS molecules	74
AI‐2 kinase	*Escherichia coli*	Brings about degradation of the autoinducers	75
Paraoxonase	Produced from wide varieties of plants, fungi, bacteria and legume	Prevents the development of biofilm by hindering the process of QS	47

### 2.6 Immobilization of enzymes

For judicious exploitation, the enzymes may be immobilized, which helps in enhancing the enzyme activity, stability, and selectivity.^49^ Immobilized enzymes show their activity over a wider range of various environmental conditions comprising of temperature, pH, and higher stability over a longer period of storage. It also helps in reducing the chances of enzyme inhibition by the reaction product, substrate, and various other components that are present in the environment.^50^ Enzyme immobilization supports in localization of enzymes as per the requirement like the fouler‐coating interface, which enhances the efficacy of the enzymes.^50^

Although free enzymes are proven to have antibiofilm efficacies, immobilization is found to increase their potential. The augmented storage stability and the reusability of the immobilized catalyst^51^ are advantageous for use as an antibiofilm and antifouling agent. Compared to free enzymes in solution, immobilized enzymes are more powerful and more resistant to environmental changes. The covalent immobilization led to the highest amount of enzyme deposited on the surface^52^ and thereby maximized the interaction between enzyme molecules and biofilm matrix.

**FIGURE 1:**
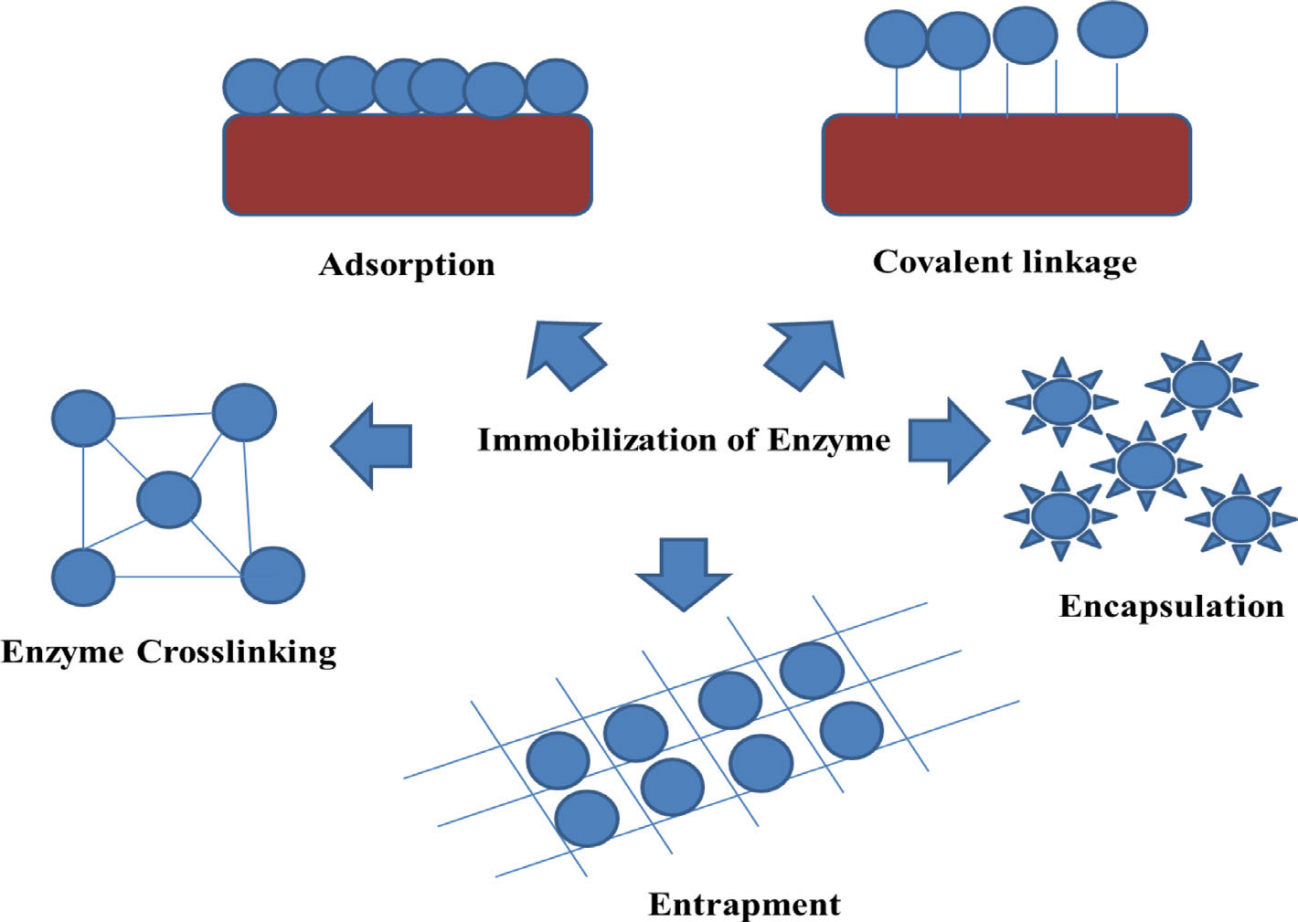
Mechanisms of enzyme immobilization

There are various types of immobilization techniques that involve covalent bonding, adsorption, graft‐copolymerization, cross‐linking, and entrapment.^53^ Generally, the mechanism of enzyme immobilization does not provide considerable results and usually trial and error mechanisms help in developing the system of immobilization needed for industrial requirement.^53^ The simplest mechanism of enzyme immobilization includes the process of entrapment and physical adsorption that result in the process of enzyme leaching from the immobilized surface, poor performance and low stability.^50^ The mechanism of covalent immobilization helps in improving the enzyme stability with minimum loss of enzyme within the aqueous media. The general mechanism of covalent immobilization help in preventing denaturation by the formation of a multiple number of covalent bonds with the enzyme followed by a reduction in the conformational flexibility. The process of thermal vibration prevents the unfolding and denaturation of the enzyme.^53^ The chemical modification brought about by covalent binding is one of the drawbacks.^53^ The retainment of the stability and activity of the enzyme results in the mechanism of site‐directed immobilization scheme by maintaining the accessibility of the active site of the enzyme to the specific substrate. The mechanism of site‐directed immobilization is an advantageous technique over the process of random immobilization^53^, ^54^ (Figure 1).

### 2.7 Advantages of enzyme immobilization

Enzymes after immobilization, show remarkable chemo as well as thermostability. It will be repeatedly used for a number of subsequent cycles. The recovery of immobilized enzyme, after a reaction becomes much easier with almost no or minimal loss the catalysts. This enhanced the cost‐effectiveness of the production process. Use of immobilized enzyme not only curtails the cost of labor, space and more, but also makes the entire handling process more convenient. Immobilization through crosslinking, entrapment, or capsulation can convert the enzyme in a form that can be applied for a variety of purposes. Hence, immobilization allows a consistent supply of products to the market. Other notable benefits of the use of immobilized enzymes in industry include enhanced efficiency of enzyme with minimized reaction time, high enzyme‐substrate ratio, and improvement in process control with less labor input. Immobilization can ameliorate the entire system with reduced opportunities for contamination in products formed.

### 2.8 Immobilized enzymes as antibiofilm agent

A number of physical adsorption or chemical entrapment matrices are used to immobilize enzymes and are found to gain more antibiofilm efficacy after immobilization (Table 2).

**TABLE 2:** The immobilization of enzymes with antibiofilm activities and their mode of action

Name of the Enzyme	Immobilization material	Mode of action as antibiofilm agent	Reference
Lysostaphin	Polydopamine is used for the purpose of immobilization that attaches the enzyme by covalent crosslinking	They inhibit the biofilm by degrading the cell wall of *Staphylococcus aureus*.	76
Ficin	Immobilized on the surface of chitosan	They help in the degradation of biofilm by *S. aureus* and enhance the susceptibility of the microbial cells to the antimicrobial agents.	77
Protease	Immobilized on the surface of chitosan	They act effectively in eradicating the biofilm formed by *Pseudomonas aeruginosa, Listeria monocytogenes* and *S. aureus*	78
Papain	Immobilized on the surface of chitosan	They effectively remove the biofilm formed by *S. aureus* and *S. epidermidis*	66
β‐N‐acetyl‐glucosaminidase	Immobilized on the surface of carboxymethyl chitosan	They effectively remove the biofilm formed by *S. aureus* and *S. epidermidis*	56
Lipase	Immobilized in polycaprolactum	Help in reducing the biofilm formed by *Escherichia coli* and *S. aureus* by bring about marked reduction of the architectural component of extracellular polymeric substances	57
Alginate lyase	Immobilized on the surface of chitosan nanoparticles	It helps in the degradation of alginate associated with the biofilm of *P. aeruginosa*.	61
DNase I	Immobilized on the surface of polydimethylsiloxane	Inhibition of the biofilm formed by *P. aeruginosa* and *S. aureus*	79
Alginate lyase	Immobilized on the surface of chitosan nanoparticles of ciprofloxacin	Helps in inhibiting the biofilm formed by *P. aeruginosa* thereby helps in preventing cystic flibrosis.	80
Hydrolase	Immobilized upon solid surface	Helps in inhibiting the biofilm formed by *E. coli*	8
Acylase	Immobilized in polyurethane	It helps in a 60% reduction in the biofilm formed by *P. aeruginosa*.	81
Cellobiose dehydrogenase	Immobilized on plasma‐activated urinary polydimethylsiloxane	Helps in the eradication of *S. aureus* biofilm	82
Lysozyme	poly(3‐hydroxybutyrate‐*co*‐3‐hydroxyhexanoate)	Helps in the eradication of the biofilm *E. coli*	83
Deoxyribonuclease I and cellobiose dehydrogenase	Surface of chitosan nanoparticles	Helps in the eradication of biofilm formed by *S. aureus* and *Candida albicans*	84
α‐Chymotryps	Immobilized on the surface of immobilized polyethylene	Helps in the eradication of biofilm formed by *E. coli*	85
Protease	Immobilization on the surface of polypropylene	Helps in the eradication of biofilm formed by *Candida albicans*	86
Glycoside Hydrolase Dispersin B	Immobilized on the surface of magnetic nanoparticles	Helps in the removal of the biofilm formed by *S. aureus*	87
Dextranase	Immobilized on the surface of the alginate	Helps in the mechanism of eradicating the biofilm formed by *Streptococcus mutans*	88
Glycoside hydrolase	Immobilized by the cross‐linking of glutaraldehyde and amine functionalization	Inhibits the formation of biofilm by *P. aeruginosa*	89
α‐amylase	Immobilized on the surface of silver nanoparticles	Helps in the purpose of eradicating the biofilm formed by multidrug resistant bacteria	90

The effects of the glycosidase pectinase and the protease subtilisin A, two commercially available immobilized enzymes were successfully applied as an antibiofilm agent against *Escherichia coli* biofilm. The best antibiofilm performance of solid‐supported hydrolases was obtained at the surface concentration of 0.022 and 0.095 U/cm^2^ with a reduction of 1.2 and 2.3 log CFU/biofilm for pectinase and subtilisin, respectively.^8^

The papain, an endolytic cysteine protease (EC: 3.4.22.2) isolated from *Carica papaya* latex immobilized on the chitosan matrixes of molecular weight (200 and 350 kDa) showed anti‐biofilm activity and increased the antimicrobials efficiency against biofilm‐embedded bacterial strains of *S. aureus* and *S. epidermidis*.^55^

The detachment of biofilm already formed by *S. epidermidis*, *S. aureus*, and *Aggregatibacter actinomycetemcomitans* was efficiently accomplished by the recombinant enzyme β‐N‐acetyl‐glucosaminidase (DspB) originally cloned from *A. actinomycetemcomitans* CU1000 and immobilized on carboxymethyl chitosan (CMCS) modified by linoleic acid (LA) after sonication.^56^

A remarkable reduction of the protein and carbohydrate content of the biofilm matrix of *S. aureus* and *S. aureus* was brought about by lipase immobilized polycaprolactam (LIP).^57^ Similarly when polycaprolactam is used to immobilize the proteolytic enzyme Subtilisin, shows antimicrobial activity against both Gram‐positive as well as negative microbes.^58^

Immobilization of subtilisin A is found to play an important role in the process of the biofilm removal.^59^ On the other hand, the enzymes, subtilisin A and the glycoside hydrolase cellulose, when immobilized through covalent crosslinking onto poly(ethylene‐alt‐maleic) anhydride copolymer films, the biofilm attachment was reduced by 44% in *P. aeruginosa*.^60^

Langumir Blodgett (LB) immobilized lipase showed about a 20% increase in antimicrobial and antibiofilm activity in comparison to its free form. Moreover, the immobilized enzyme achieved immense thermostability.^57^ A significant reduction in the carbohydrate and protein content of EPS of *S. aureus* and *E. coli* was found after treatment with lipase immobilized on polycaprolactam (LIP), a porous polymer, resembling natural polypeptide.

The immobilization of cellulase within glutaraldehyde has been found to bring about partial removal of the biofilm formed by *P. aeruginosa*.^49^

The antibiofilm efficacy of alginate lyase, Aly08, cloned from the marine bacterium *Vibrio* sp. SY01, was found to be enhanced after immobilization on low molecule weight (LMW) CS‐NPs, as the immobilized enzyme achieved more inhibitory potential for biofilm formation and eradication of mature biofilm of *P. aeruginosa*, making the bacteria more sensitive to antibiotics.^61^, ^62^, ^63^

**FIGURE 2:**
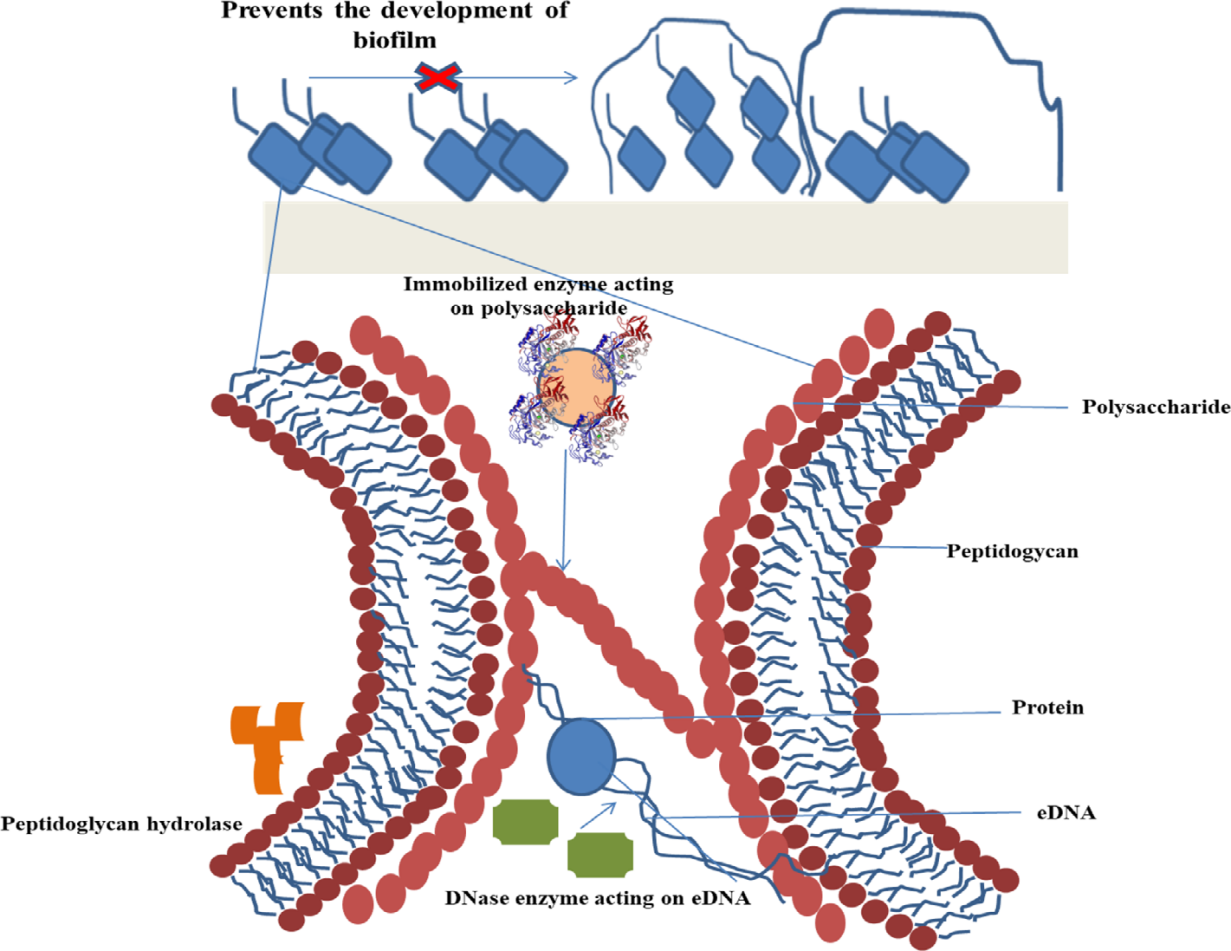
Mechanism of inhibition of biofilm by the immobilized enzyme

Amylase and Cellobiose dehydrogenase were immobilized on the surface of urinary catheters using different techniques including ultrasound, layer by layer and covalent binding, of which the maximum enzyme deposition on the surface could be achieved by covalent binding but the highest antibiofilm activities were shown when enzymes were immobilized using polyelectrolyte layer by layer technique. In this technique, enzymes are co‐assembled with polyelectrolytes and complementary functionalization with tailored protective inert polymers comprising cationic anchor groups and zwitterionic functional groups, and a coating method has been established and also successfully used for enzyme immobilization (Figure 2). Another strategy is the combination of the nonchemical modification with immobilization of CDH and/or PIPs to make an anti‐biofilm coating on the catheter.^64^

The mechanism of immobilization of lysostaphin (Lst) on the surface of polystyrene and fluorinated ethylene propylene catheters inhibits the adherence of *S. aureus* thereby preventing the formation of biofilm.^65^ It has been found that Lst‐coated equipment surfaces may be used to kill nosocomial strains of *S. aureus* in less than 15 min and prevent biofilm formation.^55^, ^66^

### 2.9 Use of nanocomposite of immobilized enzymes for treatment of biofilms

The use of nanotechnology makes the use of nanoparticles as the carriers of biocatalysts that enhances the catalytic effect due to enhanced volume to surface ratio. These nanomaterials possess the ability to immobilize various types of enzymes including lipases and cellulases that provide an innovative catalytic property.^67^, ^68^

The nanoparticles possess very high efficiency to support various types of immobilized enzymes due to its ideal characteristics for bringing about balance in the determining factors including surface area, effective enzyme loading, and mass transfer resistance. Gold nanoparticle (GNP) were developed functionalized with enzyme proteinase K (denoted as GNP + PK) and were found effective against the biofilm formed by Pseudomonas fluorescens for 72 h.^69^

The antibiofilm activity of chitosan NP are remarkably increased when linoleic acid‐modified chitosan NPs were used to immobilize the enzyme β‐N‐acetyl‐glucosaminidase (DspB) as immobilized enzyme remained active for a long time.^56^

The biofilm formed by *S. aureus* on titanium could be removed by the combination of self‐immobilization chemistry of dopamine with a biofilm‐lysing enzyme, α‐amylase, and an antimicrobial agent, silver nitrate.^70^

Nanozymes being the type of nanomaterials that possess various types of enzyme‐like properties and also exhibit various types of physicochemical properties pertaining to nanomaterials.^71^

Studies have shown protein/inorganic hybrid nanozymes by being oriented in the form of immobilized structure on the surface of inorganic grapheme nanoparticles.^72^

## 3 CONCLUSION

The recalcitrance of biofilm‐associated bacteria makes them almost impossible to tackle and the surface‐attached colonies pose a potent threat to health sectors and industries. Since almost all attempts to eradicate biofilm with conventional antibiotics are found to be ineffective, researchers are trying to explore new approaches to remove them. Among the aqueous‐soluble macromolecules, enzymes are proved to be with significant antibiofilm efficacy. In order to increase the activity, shelf life, thermostability, and reusability, the researchers prefer to immobilize the enzymes through numerous processes like adsorption, gel entrapment, covalent crosslinking, and ionotropic gelation and use them for complete eradication of biofilm. The immobilized enzymes not only inhibit biofilm formation but also can remove the already formed mature biofilm. Immobilization of enzymes amylase, cellobiohydrolase, pectinase, subtilisin A and β‐N‐acetyl‐glucosaminidase (DspB) have proved to have maximum efficiency in the eradication of the biofilm. Hence one or multiple enzymes co‐immobilized on a gel matrix may be successfully used to remove biofilm from various biotic and abiotic surfaces in a nontoxic and cost‐effective way.

## AUTHOR CONTRIBUTIONS

**Dibyajit Lahiri:** Investigation (equal). **Moupriya Nag:** Investigation (equal). **Ankita Dey:** Investigation (equal). **Tanmay Sarkar:** Investigation (equal). **Rina Rani Ray:** Investigation (equal). **Maksim Rebezov:** Investigation (equal). **Mohammad Ali Shariati:** Investigation (equal). **Muthu Thiruvengadam:** Investigation (equal). **Jesus Simal‐Gándara:** Investigation (equal).

## CONFLICT OF INTEREST

The authors declare no conflict of interest.

## Data Availability

Data sharing not applicable to this article as no datasets were generated or analyzed during the current study.

## References

[1] Muhammad MH , Idris AL , Fan X , et al. Beyond risk: bacterial biofilms and their regulating approaches. Front Microbiol. 2020;11:928. doi:10.3389/fmicb.2020.00928 32508772 PMC7253578

[2] Lahiri D , Dash S , Dutta R , Nag M . Elucidating the effect of anti‐biofilm activity of bioactive compounds extracted from plants. J Biosci. 2019;44(2):52. doi:10.1007/s12038-019-9868-4 31180065

[3] Costerton JW , Lewandowski Z , Caldwell DE , Korber DR , Lappin‐Scott HM . Microbial biofilms. Annu Rev Microbiol. 1995;49:711‐745. doi:10.1146/annurev.mi.49.100195.003431 8561477

[4] Yang L , Liu Y , Wu H , et al. Combating biofilms. FEMS Immunol Med Microbiol. 2012;65(2):146‐157. doi:10.1111/j.1574-695X.2011.00858.x 22066868

[5] Kaplan JB . Therapeutic potential of biofilm‐dispersing enzymes. Int J Artif Organs. 2009;32(9):545‐554. doi:10.1177/039139880903200903 19851978

[6] Lahiri D , Nag M , Ghosh A , et al. Biofilm and antimicrobial resistance. In: Ray RR , Nag M , Lahiri D , eds. Biofilm‐Mediated Diseases: Causes and Controls. Springer; 2021:183‐208.

[7] Lahiri D , Nag M , Sarkar T , Dutta B , Ray RR . Antibiofilm activity of α‐amylase from Bacillus subtilis and prediction of the optimized conditions for biofilm removal by response surface methodology (RSM) and artificial neural network (ANN). Appl Biochem Biotechnol. 2021;193:1853‐1872. doi:10.1007/s12010-021-03509-9 33644831

[8] Villa F , Secundo F , Polo A , Cappitelli F . Immobilized hydrolytic enzymes exhibit Antibiofilm activity against *Escherichia coli* at sub‐lethal concentrations. Curr Microbiol. 2015;71(1):106‐114. doi:10.1007/s00284-015-0834-6 25958074

[9] Lahiri D , Nag M , Dutta B , Sarkar T , Ray RR . Artificial neural network and response surface methodology‐mediated optimization of bacteriocin production by *Rhizobium leguminosarum* . Iran J Sci Technol Trans A Sci. 2021;45:1509‐1517. doi:10.1007/s40995-021-01157-6

[10] Kumar CG , Takagi H . Microbial alkaline proteases: from a bioindustrial viewpoint. Biotechnol Adv. 1999;17(7):561‐594. doi:10.1016/s0734-9750(99)00027-0 14538129

[11] Olsen SM , Pedersen LT , Laursen MH , Kiil S , Dam‐Johansen K . Enzyme‐based antifouling coatings: a review. Biofouling. 2007;23(5–6):369‐383. doi:10.1080/08927010701566384 17852071

[12] Parkar SG , Flint SH , Brooks JD . Evaluation of the effect of cleaning regimes on biofilms of thermophilic bacilli on stainless steel. J Appl Microbiol. 2004;96(1):110‐116. doi:10.1046/j.1365-2672.2003.02136.x 14678164

[13] Lahiri D , Nag M , Banerjee R , et al. Amylases: biofilm inducer or biofilm inhibitor? Front Cell Infect Microbiol. 2021;11(April):1‐13. doi:10.3389/fcimb.2021.660048 PMC8112260 33987107

[14] Masschalck B , Michiels CW . Antimicrobial properties of lysozyme in relation to foodborne vegetative bacteria. Crit Rev Microbiol. 2003;29(3):191‐214. doi:10.1080/713610448 14582617

[15] Abdou AM , Higashiguchi S , Aboueleinin AM , Kim M , Ibrahim HR . Antimicrobial peptides derived from hen egg lysozyme with inhibitory effect against *Bacillus* species. Food Control. 2007;18(2):173‐178. doi:10.1016/j.foodcont.2005.09.010

[16] Düring K , Porsch P , Mahn A , Brinkmann O , Gieffers W . The non‐enzymatic microbicidal activity of lysozymes. FEBS Lett. 1999;449(2–3):93‐100. doi:10.1016/s0014-5793(99)00405-6 10338111

[17] Garcia DL , Dillard JP . AmiC functions as an N‐acetylmuramyl‐l‐alanine amidase necessary for cell separation and can promote autolysis in Neisseria gonorrhoeae. J Bacteriol. 2006;188(20):7211‐7221. doi:10.1128/JB.00724-06 17015660 PMC1636224

[18] Rawlings N , Salvesen G . Handbook of Proteolytic Enzymes. Academic Press; 2013. doi:10.1016/C2009-1-60990-4

[19] Wu JA , Kusuma C , Mond JJ , Kokai‐Kun JF . Lysostaphin disrupts *Staphylococcus aureus* and *Staphylococcus epidermidis* biofilms on artificial surfaces. Antimicrob Agents Chemother. 2003;47(11):3407‐3414. doi:10.1128/AAC.47.11.3407-3414.2003 14576095 PMC253758

[20] Kalpana B , Aarthy S , Karutha S . Antibiofilm activity of α ‐amylase from *Bacillus subtilis* S8‐18 against biofilm forming human bacterial pathogens. Appl Biochem Biotechnol. 2012;167:1778‐1794. 22350933 10.1007/s12010-011-9526-2

[21] Muslim DS , Hasan A , Mahdi N . Antibiofilm and antiadhesive properties of pectinase purified from *Pseudomonas stutzeri* isolated from spoilt orange. Adv Environ Biol. 2016;10(11):91.

[22] Snarr BD , Baker P , Bamford NC , et al. Microbial glycoside hydrolases as antibiofilm agents with cross‐kingdom activity. Proc Natl Acad Sci. 2017;114(27):7124‐7129. doi:10.1073/pnas.1702798114 28634301 PMC5502622

[23] Tsiaprazi‐Stamou A , Monfort IY , Romani AM , Bakalis S , Gkatzionis K . The synergistic effect of enzymatic detergents on biofilm cleaning from different surfaces. Biofouling. 2019;35(8):883‐899. doi:10.1080/08927014.2019.1666108 31663364

[24] Whitchurch CB , Tolker‐Nielsen T , Ragas PC , Mattick JS . Extracellular DNA required for bacterial biofilm formation. Science. 2002;295(5559):1487. doi:10.1126/science.295.5559.1487 11859186

[25] Johansen C , Falholt P , Gram L . Enzymatic removal and disinfection of bacterial biofilms. Appl Environ Microbiol. 1997;63(9):3724‐3728. doi:10.1128/aem.63.9.3724-3728.1997 9293025 PMC168680

[26] Aldred N , Phang IY , Conlan SL , Clare AS , Vancso GJ . The effects of a serine protease, alcalase, on the adhesives of barnacle cyprids (*Balanus amphitrite*). Biofouling. 2008;24(2):97‐107. 18231899 10.1080/08927010801885908

[27] Salazar O , Asenjo JA . Enzymatic lysis of microbial cells. Biotechnol Lett. 2007;29(7):985‐994. doi:10.1007/s10529-007-9345-2 17464453

[28] Fischetti VA . Bacteriophage lytic enzymes: novel anti‐infectives. Trends Microbiol. 2005;13(10):491‐496. doi:10.1016/j.tim.2005.08.007 16125935

[29] Shehadul Islam M , Aryasomayajula A , Selvaganapathy PR . A review on macroscale and microscale cell lysis methods. Micromachines. 2017;8:83. doi:10.3390/mi8030083

[30] Park S‐Y , Kang H‐O , Jang H‐S , Lee J‐K , Koo B‐T , Yum D‐Y . Identification of extracellular N‐acylhomoserine lactone acylase from a Streptomyces sp. and its application to quorum quenching. Appl Environ Microbiol. 2005;71(5):2632‐2641. doi:10.1128/AEM.71.5.2632-2641.2005 15870355 PMC1087586

[31] Ramasubbu N , Thomas LM , Ragunath C , Kaplan JB . Structural analysis of dispersin B, a biofilm‐releasing glycoside hydrolase from the periodontopathogen Actinobacillus actinomycetemcomitans. J Mol Biol. 2005;349(3):475‐486. doi:10.1016/j.jmb.2005.03.082 15878175

[32] Izano EA , Amarante MA , Kher WB , Kaplan JB . Differential roles of poly‐N‐acetylglucosamine surface polysaccharide and extracellular DNA in *Staphylococcus aureus* and *Staphylococcus epidermidis* biofilms. Appl Environ Microbiol. 2008;74(2):470‐476. doi:10.1128/AEM.02073-07 18039822 PMC2223269

[33] Wiatr CL . Enzyme blend containing cellulase to control industrial slime 1991:1–6.

[34] Ren W , Wang S , Lü M , et al. Optimization of four types of antimicrobial agents to increase the inhibitory ability of marine *Arthrobacter oxydans* KQ11 dextranase mouthwash. Chin J Oceanol Limnol. 2016;34(2):354‐366. doi:10.1007/s00343-015-4376-3

[35] Kaplan JB , Ragunath C , Ramasubbu N , Fine DH . Detachment of *Actinobacillus actinomycetemcomitans* biofilm cells by an endogenous β‐hexosaminidase activity. J Bacteriol. 2003;185(16):4693‐4698. doi:10.1128/JB.185.16.4693-4698.2003 12896987 PMC166467

[36] Molino PJ , Wetherbee R . The biology of biofouling diatoms and their role in the development of microbial slimes. Biofouling. 2008;24(5):365‐379. doi:10.1080/08927010802254583 18604655

[37] Bonaventura C , Bonaventura J , Hooper I. Anti‐fouling methods using enzyme coatings. 1991.

[38] Dobretsov S , Dahms H‐U , Qian P‐Y . Inhibition of biofouling by marine microorganisms and their metabolites. Biofouling. 2006;22(1–2):43‐54. doi:10.1080/08927010500504784 16551560

[39] Mishra R , Panda AK , De Mandal S , Shakeel M , Bisht SS , Khan J . Natural anti‐biofilm agents: strategies to control biofilm‐forming pathogens. Front Microbiol. 2020;11:566325. doi:10.3389/fmicb.2020.566325 33193155 PMC7658412

[40] Kaplan J , Mlynek K , Hettiarachchi H , et al. Extracellular polymeric substance (EPS)‐degrading enzymes reduce staphylococcal surface attachment and biocide resistance on pig skin in vivo. PLoS One. 2018;13:e0205526. doi:10.1371/journal.pone.0205526 30304066 PMC6179274

[41] Yuan L , Hansen MF , Røder HL , Wang N , Burmølle M , He G . Mixed‐species biofilms in the food industry: current knowledge and novel control strategies. Crit Rev Food Sci Nutr. 2020;60(13):2277‐2293. doi:10.1080/10408398.2019.1632790 31257907

[42] Fetzner S . Quorum quenching enzymes. J Biotechnol. 2015;201:2‐14. doi:10.1016/j.jbiotec.2014.09.001 25220028

[43] Valko M , Leibfritz D , Moncol J , Cronin MTD , Mazur M , Telser J . Free radicals and antioxidants in normal physiological functions and human disease. Int J Biochem Cell Biol. 2007;39(1):44‐84. doi:10.1016/j.biocel.2006.07.001 16978905

[44] Klaunig JE , Kamendulis LM , Hocevar BA . Oxidative stress and oxidative damage in carcinogenesis. Toxicol Pathol. 2010;38(1):96‐109. doi:10.1177/0192623309356453 20019356

[45] Nag M , Lahiri D , Ghosh A , Das D , Ray RR . Quorum Sensing. In: Ray RR , Nag M , Lahiri D , eds. Biofilm‐Mediated Diseases: Causes and Controls. Springer; 2021.

[46] Dong YH , Xu JL , Li XZ , Zhang LH . AiiA, an enzyme that inactivates the acylhomoserine lactone quorum‐sensing signal and attenuates the virulence of *Erwinia carotovora* . Proc Natl Acad Sci U S A. 2000;97(7):3526‐3531. doi:10.1073/pnas.060023897 10716724 PMC16273

[47] Ozer EA , Pezzulo A , Shih DM , et al. Human and murine paraoxonase 1 are host modulators of *Pseudomonas aeruginosa* quorum‐sensing. FEMS Microbiol Lett. 2005;253(1):29‐37. doi:10.1016/j.femsle.2005.09.023 16260097

[48] Rothfork JM , Timmins GS , Harris MN , et al. Inactivation of a bacterial virulence pheromone by phagocyte‐derived oxidants: new role for the NADPH oxidase in host defense. Proc Natl Acad Sci U S A. 2004;101(38):13867‐13872. doi:10.1073/pnas.0402996101 15353593 PMC518845

[49] Cordeiro AL , Werner C . Enzymes for antifouling strategies. J Adhes Sci Technol. 2011;25(17):2317‐2344. doi:10.1163/016942411X574961

[50] Cao L . Carrier‐Bound Immobilized Enzymes: Principles, Application and Design. Wiley; 2005. doi:10.1002/3527607668

[51] Dulik DM , Fenselau C . Use of immobilized enzymes in drug metabolism studies. FASEB J. 1988;2(7):2235‐2240. doi:10.1096/fasebj.2.7.3127263 3127263

[52] Vergidis P , Patel R . Novel approaches to the diagnosis, prevention, and treatment of medical device‐associated infections. Infect Dis Clin N Am. 2012;26(1):173‐186. doi:10.1016/j.idc.2011.09.012 PMC3269005 22284383

[53] Hanefeld U , Gardossi L , Magner E . Understanding enzyme immobilisation. Chem Soc Rev. 2009;38(2):453‐468. doi:10.1039/b711564b 19169460

[54] Viswanath S , Wang J , Bachas LG , Butterfield DA , Bhattacharyya D . Site‐directed and random immobilization of subtilisin on functionalized membranes: activity determination in aqueous and organic media. Biotechnol Bioeng. 1998;60(5):608‐616. 10099469

[55] Baidamshina DR , Koroleva VA , Olshannikova SS , et al. Biochemical properties and anti‐biofilm activity of chitosan‐immobilized papain. Mar Drugs. 2021;19(4):197. doi:10.3390/md19040197 33807362 PMC8066807

[56] Tan Y , Ma S , Liu C , Yu W , Han F . Enhancing the stability and antibiofilm activity of DspB by immobilization on carboxymethyl chitosan nanoparticles. Microbiol Res. 2015;178:35‐41. doi:10.1016/j.micres.2015.06.001 26302845

[57] Prabhawathi V , Boobalan T , Sivakumar PM , Doble M . Antibiofilm properties of interfacially active lipase immobilized porous polycaprolactam prepared by LB technique. PLoS One. 2014;9(5):e96152. doi:10.1371/journal.pone.0096152 24798482 PMC4010425

[58] Veluchamy P , Sivakumar PM , Doble M . Immobilization of subtilisin on polycaprolactam for antimicrobial food packaging applications. J Agric Food Chem. 2011;59(20):10869‐10878. doi:10.1021/jf201124v 21910484

[59] Shiah J‐G . Bioconjugation protocols. Strategies and methods. Volume 283 in methods in molecular biol‐ogy edited by Christof M. Niemeyer. Humana Press, Totowa, NJ. 2004. xi + 330 pp. 16 × 24 cm. ISBN 1‐588‐29‐098‐0. $125.00. J Med Chem. 2004;47(25):6434. doi:10.1021/jm0401715

[60] Cordeiro AL , Hippius C , Werner C . Immobilized enzymes affect biofilm formation. Biotechnol Lett. 2011;33(9):1897‐1904. doi:10.1007/s10529-011-0643-3 21618024

[61] Li S , Wang Y , Li X , Lee BS , Jung S , Lee M‐S . Enhancing the thermo‐stability and anti‐biofilm activity of alginate lyase by immobilization on low molecular weight chitosan nanoparticles. Int J Mol Sci. 2019;20(18):4565. doi:10.3390/ijms20184565 31540110 PMC6770906

[62] Kristensen JB , Meyer RL , Laursen BS , Shipovskov S , Besenbacher F , Poulsen CH . Antifouling enzymes and the biochemistry of marine settlement. Biotechnol Adv. 2008;26(5):471‐481. doi:10.1016/j.biotechadv.2008.05.005 18619758

[63] Nuhiji E , Wong CS , Sutti A , Lin T , Kirkland M , Wang X . Biofunctionalization of 3D nylon 6,6 scaffolds using a two‐step surface modification. ACS Appl Mater Interfaces. 2012;4(6):2912‐2919. doi:10.1021/am300087k 22663066

[64] Wi YM , Patel R . Understanding biofilms and novel approaches to the diagnosis, prevention, and treatment of medical device‐associated infections. Infect Dis Clin N Am. 2018;32(4):915‐929. doi:10.1016/j.idc.2018.06.009 PMC6215726 30241715

[65] Shah A , Mond J , Walsh S . Lysostaphin‐coated catheters eradicate *Staphylococccus aureus* challenge and block surface colonization. Antimicrob Agents Chemother. 2004;48(7):2704‐2707. doi:10.1128/AAC.48.7.2704-2707.2004 15215130 PMC434171

[66] Trizna EY , Baydamshina DR , Kholyavka MG , et al. Soluble and immobilized papain and trypsin as destroyers of bacterial biofilms. Genes Cells. 2015;10(3):106‐112.

[67] Singh N , Dhanya BS , Verma ML . Nano‐immobilized biocatalysts and their potential biotechnological applications in bioenergy production. Mater Sci Energy Technol. 2020;3:808‐824. doi:10.1016/j.mset.2020.09.006

[68] Lahiri D , Nag M , Sheikh HI , et al. Microbiologically synthesized nanoparticles and their role in silencing the biofilm signaling cascade. Front Microbiol. 2021;12:636588. doi:10.3389/fmicb.2021.636588 33717030 PMC7947885

[69] Habimana O , Zanoni M , Vitale S , et al. One particle, two targets: a combined action of functionalised gold nanoparticles, against Pseudomonas fluorescens biofilms. J Colloid Interface Sci. 2018;526:419‐428. doi:10.1016/j.jcis.2018.05.014 29763820

[70] Tran HA , Tran PA . Immobilization‐enhanced eradication of bacterial biofilms and in situ antimicrobial coating of implant material surface ‐ an in vitro study. Int J Nanomedicine. 2019;14:9351‐9360. doi:10.2147/IJN.S219487 31819436 PMC6890190

[71] Wang P , Wang T , Hong J , Yan X , Liang M . Nanozymes: a new disease imaging strategy. Front Bioeng Biotechnol. 2020;8:15. doi:10.3389/fbioe.2020.00015 32117909 PMC7015899

[72] Liu Y , Zhu G , Bao C , Yuan A , Shen X . Intrinsic peroxidase‐like activity of porous CuO micro−/nanostructures with clean surface. Chin J Chem. 2014;32(2):151‐156. doi:10.1002/cjoc.201300683

[73] Lade H , Paul D , Kweon JH . Quorum quenching mediated approaches for control of membrane biofouling. Int J Biol Sci. 2014;10(5):550‐565. doi:10.7150/ijbs.9028 24910534 PMC4046882

[74] Pustelny C , Albers A , Büldt‐Karentzopoulos K , et al. Dioxygenase‐mediated quenching of quinolone‐dependent quorum sensing in *Pseudomonas aeruginosa* . Chem Biol. 2009;16(12):1259‐1267. doi:10.1016/j.chembiol.2009.11.013 20064436

[75] Roy V , Fernandes R , Tsao C‐Y , Bentley WE . Cross species quorum quenching using a native AI‐2 processing enzyme. ACS Chem Biol. 2010;5(2):223‐232. doi:10.1021/cb9002738 20025244

[76] Yeroslavsky G , Girshevitz O , Foster‐Frey J , Donovan DM , Rahimipour S . Antibacterial and Antibiofilm surfaces through Polydopamine‐assisted immobilization of Lysostaphin as an antibacterial enzyme. Langmuir. 2015;31(3):1064‐1073. doi:10.1021/la503911m 25547537

[77] Baidamshina DR , Koroleva VA , Trizna EY , et al. Anti‐biofilm and wound‐healing activity of chitosan‐immobilized Ficin. Int J Biol Macromol. 2020;164:4205‐4217. doi:10.1016/j.ijbiomac.2020.09.030 32916198

[78] Elchinger P‐H , Delattre C , Faure S , et al. Immobilization of proteases on chitosan for the development of films with anti‐biofilm properties. Int J Biol Macromol. 2015;72:1063‐1068. doi:10.1016/j.ijbiomac.2014.09.061 25451753

[79] Alves D , Magalhães A , Grzywacz D , Neubauer D , Kamysz W , Pereira MO . Co‐immobilization of palm and DNase I for the development of an effective anti‐infective coating for catheter surfaces. Acta Biomater. 2016;44:313‐322. doi:10.1016/j.actbio.2016.08.010 27514277

[80] Patel KK , Tripathi M , Pandey N , et al. Alginate lyase immobilized chitosan nanoparticles of ciprofloxacin for the improved antimicrobial activity against the biofilm associated mucoid *P. aeruginosa* infection in cystic fibrosis. Int J Pharm. 2019;563:30‐42. doi:10.1016/j.ijpharm.2019.03.051 30926526

[81] Grover N , Plaks JG , Summers SR , Chado GR , Schurr MJ , Kaar JL . Acylase‐containing polyurethane coatings with anti‐biofilm activity. Biotechnol Bioeng. 2016;113(12):2535‐2543. doi:10.1002/bit.26019 27240552

[82] Thallinger B , Brandauer M , Burger P , et al. Cellobiose dehydrogenase functionalized urinary catheter as novel antibiofilm system. J Biomed Mater Res Part B Appl Biomater. 2016;104(7):1448‐1456. doi:10.1002/jbm.b.33491 26251187

[83] Kehail AA , Brigham CJ . Anti‐biofilm activity of solvent‐cast and electrospun polyhydroxyalkanoate membranes treated with lysozyme. J Polym Environ. 2018;26(1):66‐72. doi:10.1007/s10924-016-0921-1

[84] Tan Y , Ma S , Leonhard M , Moser D , Ludwig R , Schneider‐Stickler B . Co‐immobilization of cellobiose dehydrogenase and deoxyribonuclease I on chitosan nanoparticles against fungal/bacterial polymicrobial biofilms targeting both biofilm matrix and microorganisms. Mater Sci Eng C. 2020;108:110499. doi:10.1016/j.msec.2019.110499 31923978

[85] Cattò C , Secundo F , James G , Villa F , Cappitelli F . α‐Chymotrypsin immobilized on a low‐density polyethylene surface successfully weakens *Escherichia coli* biofilm formation. Int J Mol Sci. 2018;19(12): 4003. doi:10.3390/ijms19124003 30545074 PMC6321288

[86] Andreani ES , Villa F , Cappitelli F , et al. Coating polypropylene surfaces with protease weakens the adhesion and increases the dispersion of *Candida albicans* cells. Biotechnol Lett. 2017;39(3):423‐428. doi:10.1007/s10529-016-2262-5 27878654

[87] Liu Z , Zhao Z , Zeng K , et al. Functional immobilization of a biofilm‐releasing glycoside hydrolase dispersin B on magnetic nanoparticles. Appl Biochem Biotechnol. 2021;194:737‐747. doi:10.1007/s12010-021-03673-y 34524634

[88] Juntarachot N , Sirilun S , Kantachote D , et al. Anti‐*Streptococcus mutans* and anti‐biofilm activities of dextranase and its encapsulation in alginate beads for application in toothpaste. PeerJ. 2020;8:e10165. doi:10.7717/peerj.10165 33240599 PMC7678491

[89] Asker D , Awad TS , Baker P , Howell PL , Hatton BD . Non‐eluting, surface‐bound enzymes disrupt surface attachment of bacteria by continuous biofilm polysaccharide degradation. Biomaterials. 2018;167:168‐176. doi:10.1016/j.biomaterials.2018.03.016 29571052

[90] Abeleda HEP , Javier AP , Murillo AQM , Baculi RQ . Alpha‐amylase conjugated biogenic silver nanoparticles as innovative strategy against biofilm‐forming multidrug resistant bacteria. Biocatal Agric Biotechnol. 2020;29:101784. doi:10.1016/j.bcab.2020.101784

